# Editorial: The Next Step in Developmental Embodiment Research: Integrating Concepts and Methods

**DOI:** 10.3389/fnsys.2022.871449

**Published:** 2022-03-23

**Authors:** Vanessa Lux, Gustaf Gredebäck, Amy L. Non, Melanie Krüger

**Affiliations:** ^1^Department of Genetic Psychology, Ruhr University Bochum, Bochum, Germany; ^2^Department of Psychology, Uppsala University, Uppsala, Sweden; ^3^Department of Anthropology, University of California San Diego, La Jolla, CA, United States; ^4^Institute of Sports Science, Leibniz University Hannover, Hannover, Germany

**Keywords:** embodiment, lifespan development, epigenetics, interoception, embodied cognition

## Introduction

Embodiment has become a key concept in human life sciences, and specifically in the study of the human mind. The question, how to integrate a developmental perspective with embodiment research, has recently emerged in cognitive science (Maruyama et al., 2014; Nava et al., 2018; Marmeleira and Duarte Santos, 2019), developmental psychology (Marshall, 2014; Gredebäck and Falck-Ytter, 2015), language processing studies (Dove, 2018), and robotics (Gordon, 2019; Kuniyoshi, 2019). In addition, calls for expanding the timeframe of developmental processes under study beyond early childhood to the lifespan are voiced (Loeffler et al., 2016; Reed et al., 2019). However, these first attempts emerged separately in each of their respective fields.

The aim of this Research Topic was to overcome this divide by inviting theoretical and empirical contributions targeting the developmental dynamics of embodiment phenomena across a broad range of disciplines. We invited contributions that discuss different timescales of developmental dynamics—from sensitive periods to across the lifespan, intergenerational, and longer-scale evolutionary perspectives—along with contributions that target different system levels—from epigenetics to complex motor behavior.

## Theoretical Perspectives on Developmental Embodiment Research

In a thematic review, Lux et al. propose to integrate embodiment approaches from a developmental perspective. They distinguish between two lines of embodiment approaches: environmental approaches which focus on the biological embedding of experiences, specifically in sensitive periods of development, and agency approaches, which emphasize the role of embodiment in active interaction with the environment. In addition, they outline different levels of embodiment, related data modalities, and the timescales for which developmental embodiment research needs to account. They argue that the study of embodiment phenomena needs to be function-specific, cross-level, and accounting for the different timescales of developmental processes thus implying an interdisciplinary approach.

In his opinion paper, Lickliter critically examines the proposal by Lux et al. and highlights two important issues for developmental embodiment research: First, from a developmental perspective, the mechanisms and dynamics which bring about phenotypic stability and phenotypic variability are the same. Studying them in the context of an individual's active engagement with the environment allows one to pinpoint change and stability along the specific developmental trajectory. Second, embodiment opens up and at the same time constrains possible developmental outcomes, but this opening-up and constraint is itself a result of development. Thus, a developmental perspective clearly illuminates the mechanisms underlying embodiment phenomena, but at the same time points to the complexity of this interdisciplinary endeavor.

Coming from the perspective of developmental science, Marshall et al. argue that an integration of developmental and embodiment research needs to account for three different epistemological states of the body: the body as biological organism, addressed by phylo- and ontogenetic developmental theories, the body as medium for the interaction with the environment, which emphasizes an interactionist and co-constructivist approach, and the body as lived experience, which points toward the integration of phenomenological accounts. They advocate for placing the study of organization and systems in the center of developmental science and adapting the metatheoretical framework of process-relational developmental systems theory (e.g., Overton and Lerner, 2014).

While these articles highlight the interdisciplinarity of developmental embodiment research, Mille et al. outline the potential within a specific research field: the study of cognitive changes associated with aging. They explore how age-related changes in the neurophysiological substrates underpinning perceptual and memory interactions in older adults could explain the more broad cognitive changes associated with aging in terms of gains and losses.

## Methodological Accounts For A Developmental Embodiment Perspective

Another set of contributions introduce new experimental paradigms to study the interrelatedness of embodiment and development or re-interpret existing paradigms, data, and methods from a developmental embodiment perspective.

In their thematic review, Sen and Gredebäck introduce an embodied account for the “mobile paradigm” (Rovee and Rovee, 1969; Rovee-Collier, 1995), a well-established approach to study the development of memory skills through operant conditioning of simple motor behavior in infants. According to Sen and Gredebäck, an embodied account of the mobile paradigm accounts for the individual sensorimotor experiences—and importantly their interindividual variance—gained from the motor interaction with the environment. Thus, this approach would be more powerful in comprehensively explaining developmental changes in cognitive and motor functions.

Similarly, Musculus et al. advocate for an embodied approach for the investigation of developmental changes in action planning across the lifespan. Based on an in-depth review of research on motor and cognitive planning, they argue that cognitive development is fundamentally driven and constrained by motor development and needs to be studied in an integrated manner. For this, they propose the integrative concept of “embodied planning” and introduce a novel climbing paradigm to study action planning from a developmental embodiment perspective.

In their contribution, Riva et al. propose a new therapeutic method, “Regenerative Virtual Therapy,” to investigate and intervene in the dynamics of a faulty bodily self-consciousness underlying mental disorders. Using a Bayesian modeling approach, they suggest combining mindful attention, cognitive reappraisal, and brain stimulation techniques with rewarding multisensory bodily experiences may overwrite faulty bodily experiences and regenerate wellbeing.

## Developmental Embodiment Research: Empirical Studies

Five articles contribute original research studying different levels of embodiment from a developmental perspective. Reggin et al. investigated abstract word acquisition from a linguistic developmental perspective and found partial support for the affective embodiment account: Word valence, interoception, and mouth action facilitated abstract word acquisition more than concrete word acquisition. They conclude that affective embodiment is important to children's acquisition of abstract words, but that there is considerable variance to be accounted for by other factors such as contextual diversity of vocabulary use. Wienecke et al. tested whether a combination of basketball training and mathematics improves motivation for classroom-based mathematics in school children. Their findings indicate that learning mathematics in combination with physical activity increases intrinsic motivation levels while the underlying processes still warrant future investigations.

In contrast to these studies, Amico and Schaefer report findings that walking toward the target during a visual-spatial working memory task impaired encoding and recall performance across age groups. Their results clearly contradict earlier studies in the field of cognitive embodiment and, thus, support the notion that embodiment processes are highly functional specific.

Finally, two papers present research conducted in populations of particular interest for developmental embodiment research: highly functional adults and vulnerable groups. Stadler et al. studied shared action representations in Taekwondo experts and novices using video-based evaluations of complex movement sequences. They show significantly more overlap within the expert group as compared to the control group. They conclude that experts might benefit from sensorimotor skills to simulate the observed actions more precisely and that this enhances shared representations. Clausing and Non investigated whether immigration-related stress impacts the cardiometabolic risk and epigenetic markers of Latinx immigrant mothers and their children, integrating a cross-level approach with an intergenerational perspective. They found associations of stress markers with cardiometabolic risk and, to a smaller degree, with epigenetic markers indicating that the life circumstances of immigrant families can become biologically embedded in both adults and children, and that DNA methylation may be on the pathway linking stress with cardiometabolic risk.

## Conclusion

Overall, the contributions highlight the growing relevance and potential of developmental embodiment research. In some cases, this approach allowed integration of contradictory findings, in others it challenged existing knowledge or opened-up new research avenues. Also, the framework requires methodological innovations which explicitly target cross-level dependencies—some of which are presented here. The research articles presented in this special issue highlight the value of such cross-level examinations and the potential of focusing on sensitive periods, comparisons across the lifespan, and specific populations to further elucidate the developmental dynamics of embodiment processes. Developmental embodiment research has clearly taken the next step.

## Author Contributions

VL and MK drafted the first version of the manuscript. All authors contributed to, commented on previous versions of the manuscript, read, and approved the final manuscript.

## Conflict of Interest

The authors declare that the research was conducted in the absence of any commercial or financial relationships that could be construed as a potential conflict of interest.

## Publisher's Note

All claims expressed in this article are solely those of the authors and do not necessarily represent those of their affiliated organizations, or those of the publisher, the editors and the reviewers. Any product that may be evaluated in this article, or claim that may be made by its manufacturer, is not guaranteed or endorsed by the publisher.
